# Distributed Estimation Techniques for Cyber-Physical Systems: A Systematic Review

**DOI:** 10.3390/s19214720

**Published:** 2019-10-30

**Authors:** Carmelina Ierardi, Luis Orihuela, Isabel Jurado

**Affiliations:** Department of Engineering, Universidad Loyola Andalucía, 41704 Seville, Spain; dorihuela@uloyola.es (L.O.); ijurado@uloyola.es (I.J.)

**Keywords:** cyber-physical systems, distributed estimation, systematic review

## Abstract

This paper undertakes a systematic review (SR) on distributed estimation techniques applied to cyber-physical systems (CPS). Even though SRs are not the common way to survey a theme in the control community, they provide a rigorous, robust and objective formula that should not be always ignored. The presented SR incorporates and adapts the guidelines recommended in other fields (mainly biosciences and computer sciences) to the field of automation and control and presents a brief description of the different phases that constitute an SR. As a result, this review compares the different techniques found in the literature in terms of: The proposed estimator (Kalman filter, Luenberger observer, Bayesian filter, etc.), the particular application within CPS, the design of the estimators (decentralized vs centralized), the amount of data required for implementation or the inclusion of experiments/simulations in the studies. Particular attention is paid to those papers that present some results in applications that include humans, animals or biological systems.

## 1. Introduction

The need to undertake a thorough and complete review on a given topic arises to reinforce the basic knowledge and, above all, the evolution and improvements that have taken place over the years. The large amount of information available makes it surprisingly difficult to review a precise research topic in an appropriate manner. Some of the common issues are related to the subjectivity of the reviewer, the lack of normalisation between the authors or the way in which the databases are searched. Hence, a systematic review (SR) or sometimes called systematic literature review, arises as a possible solution, since it has been applied with great success in other fields.

An SR is a form of secondary study, whereas individual studies, which contribute to the systematic review itself, are termed primary studies [1]. An SR focuses on one or more specific research question on the topic to be treated, using only primary studies related to the subject. The thematic areas that already use this kind of review range from medicine to economics, from psychology to software engineering. However, in our area of interest, automation and control, traditional survey papers are dominant. These narrative reviews are usually performed by researchers with extensive experience and knowledge in the field, providing their global or general view of the topic, see for instance [2,3,4]. While the latter is characterised by exploiting the experts’ knowledge and their broad point of view, the former is distinguished by its objectivity and rigorousness.

In this paper, we inherit and adjust the good practices described in the bio-science [5,6] and computer science [1,7,8] literature to the particular features of our field. In addition, we adapt the PRISMA method (preferred reporting items for systematic reviews and meta-analyses) [9], to establish fundamental procedures to draw up the SR. This constitutes the first goal of the paper.

As the second and main objective, this paper aims to apply this methodology in order to review the distributed estimation techniques that have been applied to cyber-physical systems (CPS). Cyber-physical systems are complex systems composed of entities of different natures that interact with a given physical medium [10]. They can simultaneously have communication, computation and control capabilities and they can involve humans, animals and biological process [3]. Distributed estimation techniques aim to know the inner state of a system by using the information provided by the measurements locally collected from the plant and that information interchanged with the rest of agents [11].

To be more precise, we are interested in those heterogeneous systems that incorporate a physical part and a cybernetic layer. We are focused on the estimation of dynamical systems, so those papers that propose static estimators, such as those referred to in [12], are not included in the review. Another key feature of this SR is the fact that the distributed estimators must exchange some sort of information with other agents, in such a way that pure decentralised schemes (without communication) or sensor fusion techniques (all the information is gathered at a single node) are excluded. These constitute, mainly, the inclusion criteria for the studies appearing in the review.

After screening more than 2800 possible papers, only 20 primary studies satisfy the aforementioned criteria. We have carefully revised those papers and have collected the following data: The sort of estimator used (Kalman filter, Luenberger observer, Bayesian filter, etc.), the design of the estimators (if decentralised or centralised), the amount of data that must be exchanged between agents, the communication protocol (all-to-all, scheduled, just with neighbors, etc.), the particular application, the inclusion of experiments or simulations and some other advantages/disadvantages. Special focus has been put on those studies that apply these methodologies to humans, animals or biological systems. In the authors’ opinion, the inclusion of such systems in the estimation loop hinders the design/performance of the observers, demanding particular attention. All this information, gathered in a feature table, is very useful to get a complete, rigorous and objective view of the revised topic. Finally, the research questions initially formulated are answered trying to provide a full and objective perspective of the topic.

This paper is organised as follows. Section 2 presents the preliminary concepts and the description of the different phases of the SR. The report of the systematic review is given in Section 3. Finally, the conclusions are drawn in Section 4.

## 2. Preliminary Concepts

A proper bibliographic review appears to be the necessary first step for every research, aiming to get deep insight into the state of the art to understand how it has evolved over the years and at what point it is nowadays.

This paper intends to bring the methodology of systematic reviews to the field of automation and control, using as guides published SRs in other areas of the literature, mainly social sciences and software engineering [1,6,7,8,13].

**Figure 1:**
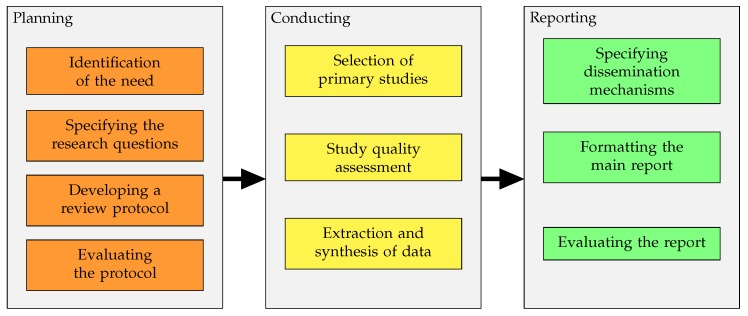
Phases y sub-phases of the systematic review [1].

Typically, a systematic review of the literature consists of three well-defined sequential phases: The planning, the conducting and the reporting, as shown in Figure 1. In the next subsections, we will adapt this methodology to the peculiarities of our field.

### 2.1. Description of Phase 1: Planning the Review

The first step is the planning, perhaps the most important because it is the basis of the whole review. It is divided into four actions and it begins with the identification of the need for a systematic review. The researchers should identify existing systematic—which are almost inexistent in our field—and traditional reviews on the topic and ensure if it is worth undertaking the review.

The most important part of any SR is the formulation of one or more specific questions to which the SR expects to respond. As it is explained in [1], a right question should be meaningful to both practitioners and researchers. An adequate question should suggest modifications or increase the confidence on the current practice. A correct research of what we want to look for is fundamental for a targeted research, eliminating redundant information.

There are some guidelines for the formulation of the research questions in the medical field [14] or software engineering [1], but none on automation and control. It has been suggested to use the PICOC criteria (population, intervention, comparison, outcome, context) to frame research questions in social sciences [15] and in computer science [1]. Next, we make a reflection on how these criteria can be particularized to the automation and control sciences:

**Population:** The target population could be very varied:

- An application area, such as, solar fields, traffic systems, biological systems or smart grids.
- A particular description of a dynamical system, such as, linear vs nonlinear differential equations, state-space vs transfer functions, with or without noises or disturbances, uncertainty models, centralised vs distributed, etc.
- A well-described framework, such as, fault detection, formation control, networked control systems, system identification, etc.
- A specific course, module, group of students, etc.
- A given technology or equipment, as for instance an autonomous car, a chemical plant, a robotic arm or a drone.

Even, the population could consist of a combination of the mentioned items.

**Intervention:** The intervention is the particular automation and control methodology/algorithm/ technology that addresses a specific task. For instance, it could be a model predictive control algorithm, a methodology for modeling robotic systems or a technology for performing hardware-in-the-loop simulations.

**Comparison:** It is the particular automation and control methodology/algorithm/technology that serves as comparison. For instance, distributed methodologies could be compared to centralised ones or linear controllers could be compared with standard PID controllers.

**Outcome:** These are factors of importance to practitioners and researchers that the proposed intervention achieves. This might be difficult to make precise, because there does not exist a normalisation of the outcomes of the studies in the control community. This drawback, which is also common for computer scientist, does not appear in social sciences, since their outcomes follow certain rules.

Some standards that usually appear in our field are: Qualitative/quantitative performance, rejection of disturbances/noises, reliability, resiliency to attacks, robustness against uncertainties in the models, computational complexity, communication needs, energy requirements or fulfilment of real-time needs.

**Context:** This is the context in which the comparison takes place. In this field, researchers make use of experiments or simulations to make comparisons with available interventions. Hence, some possible criteria could be: The presence/absence of a comparison, numerical vs applied simulations, field experiments, etc.

Please note that some of the previous criteria, such as outcome or context, are general and, therefore, equivalent to other fields. We have included them here in order to present a self contained document. Moreover, several examples, with standard keywords, have been provided to help the potential reader in the application of this criteria to automation and control.

Once the research questions have been formulated, the next step consists in developing and, later, evaluating a protocol. The protocol mainly serves to reduce the partiality of the study, defining clearly and precociously how to conduct the entire process of the systematic review, with every situation and norm to be followed in each phase and sub-phase [1,13,15]. In bio-sciences, the protocol is sometimes registered in a prospective register, such as PROSPERO (https://www.crd.york.ac.uk/prospero/). However, these kinds of registers do not exist in the automation and control field.

A protocol is usually organised in three main parts, namely, introduction, methodology and a brief discussion. The introduction presents the background and context of the particular area, stating the need and aims of the review, with the research questions.

The methodology should be based in a predefined standard guide, such as the PRISMA guidelines for SRs [9]. Based on those guidelines, the methodology should include, at least, the following items:

- Search strategy, that is, the key terms for the search, normally written as a Boolean function. In addition, it must be stated the sources that are to be searched to find the studies, such as digital libraries, specific journals and conference proceedings and the databases that include the contents of those sources.
- The inclusion and exclusion criteria for the studies.
- The particular procedure to be followed for the selection of primary studies. In particular, it should be mentioned the number of researchers that will evaluate the documents and the way in which a possible disagreement will be resolved.
- Assessment of risk of bias using, if possible, a standard method, such as the Cochrane guidelines [16].
- Strategy for data extraction, clearly identifying the information that is going to be gathered from the included primary studies.

It is a common practice in medicine to submit the protocol to peer review. In automation and control, it could be interesting to submit the protocol to a peer-reviewed conference which a certain impact, such us the CDC, ACC, IFAC WC or ECC. This could serve as a good evaluation of the protocol. In addition and since SR are almost inexistent in automation and control, the review protocol could be evaluated by an independent expert in bio-sciences or software.

**Particular Features for the Methodology in Automation and Control**

In this section, we will provide some notes concerning the methodology in the automation and control field.

Initially, the search begins by defining the Boolean function, which is nothing more than an extraction of keywords, joined by the supported operators, Booleans or not, from the different databases. We can also define it as a string with the most important words and synonyms extrapolated from the initial research questions. At this point, it is worth revising several survey papers and key studies in the field to find all the possible terminology associated to the research questions.

The next step is to appropriately choose the databases best suited to the topic of investigation, making sure to get a good coverage of the most important publishers. From the authors’ point of view, in automation and control, the most important databases to be considered for a review are presented in Table 1. According to the information that these databases put in proportion (https://webofknowledge.com/; https://dl.acm.org/; https://ieeexplore.ieee.org/; https://www.scopus.com/; https://www.sciencedirect.com/; https://link.springer.com/; https://onlinelibrary.wiley.com/), most of the magazines and journals in automation and control and also the main conferences in this area, are published by publishers whose content appears in those databases.

Table 1 uses some acronyms to indicate the search fields. The most common searches are the one that covers the fields of abstracts, titles and keywords of the paper, labeled with (A+T+K). Other characteristics are also included in Table 1, such as the number of maximum citations that can be downloaded at a single step, the format in which the citations can be downloaded, whether the downloads include the abstract or not, the Boolean operators that can be used (each database uses its own characters and Boolean operators) and the maximum number of terms allowed in the search string. Furthermore, there are two ways to perform the search in the databases: A structured advanced search (automatic) and a manual search by writing suitable commands (manual). In general, the last one let us perform more complex searches.

Another important step of the SR is the establishment of the inclusion and exclusion criteria, which are fundamental in the selection process of the papers. Each candidate study, in order to move to the next stage, must include all inclusion criteria and must not present any of the exclusion criteria. The criteria, as indeed every step of the review, must be very clear, because the selection of primary studies is carried out by two people at the same time, who evaluate each work separately. In order to be included in the SR, the primary study must be accepted by both reviewers. In medicine or psychology, this evaluation is normally done by reading Title and Abstract. However, as it has been discovered by conducting this SR, titles an abstract in automation and control and other engineering fields are seldom normalised and, unluckily, sometimes they do not contain the necessary information to perform the evaluation. In those cases, the reviewer must read the full text. In the case of disagreement, a third person will decide whether to include it or not. This is a well-extended practice in SRs that can be inherited here.

The last points to consider are the study quality assessment checklist and the data extraction strategy. As we have already seen with other aspects of the SR, there does not exist a quality checklist to assess the individual studies in our field, so the reviewers could opt between modifying and adapt those found in the bio-science/social science literature or discarding this step.

Finally, for the data extraction, the following list of items is suggested. When extracting the data from the selected papers, among the characteristics that could be extracted, in general there are:

- Basic information: Authors, year of publication, title, etc.
- Field of application or study subjects or study type of each paper.
- Particular information related to the application context.
- Tools used.
- Test/experiment/simulation.
- Limitations and advantages.

### 2.2. Description of Phase 2: Conducting the Review

Once the precise research questions have been established, the appropriate databases have been chosen, the Boolean function has been defined and the inclusion and exclusion criteria have been set, we are ready to move on to the second block of the SR, presented in the Figure 1, which basically includes the selection of primary studies, study quality assessment, extraction and synthesis of data.

In order to guide this process, an international group of experts has developed the PRISMA method: A framework for the realisation of SR [9], which consists of 27 points and a flow diagram (see Section 3) that summarises the selection process.

At this point, the reviewers are faced with a great quantity of results, coming from the different databases used. The first filter to be applied, which is often included directly in the database, is the elimination of gray literature, which includes books, book chapters, poster presentations, reviews, surveys and doctoral theses that usually give rise to a journal article. In conclusion, we will include only original works that have gone through a peer-review process. It is of a crucial importance to use a tool to eliminate the duplicates, since the databases in automation and control share part of their content.

Then, the obtained primary studies will be evaluated by the reviewers (normally two people, as explained before) following the predefined protocol and applying the inclusion and exclusion criteria. This step will provide us the list of selected primary studies to be included in the SR.

In order to improve the list of studies, it is a good practice at this stage to contact the authors of the selected papers and check their bibliography to see if any possible paper has escaped the initial search. These additional papers will be included in the list of selected primary studies under the name of additional records identified through other sources (see Section 3). In the event that many items were found that were not included in the first list of studies, it is advisable to restart the SR, following the PRISMA procedure again.

The quality of the different works is evaluated using the tool presented in this subsection. For this paper, we have developed a very simple checklist that can be used to assess the quality of the papers included in the SR, see Table 2.

For the extraction and synthesis of the data of each selected paper one can resort to different ways to re-organise the information, such as a narrative synthesis, a concept maps or a table of characteristics. In the authors’ opinion, the latter is, perhaps, more useful in automation and control, since the reader (practitioner or researcher) will easily see the information collected from the studies and will be able to extract his/her own conclusions. These tables, in addition to standard information such as authors and year of publication, present the information that has been previously stated in the protocol. They summarise in a visual way the features and peculiarities of each work.

The data extraction procedure should be performed by more than one reviewer, following a similar protocol than the one established for the inclusion of primary studies. If this cannot be done, at least, some methods should be introduced to check that the data are being extracted correctly. For instance, if a PhD is conducting this phase, the supervisor could randomly pick some of the studies and extract the features, to make a comparison with the data extracted by the student. In general, the risk of bias or rigorousness can be reduced by including more people in any decision.

### 2.3. Description of Phase 3: Reporting the Review

The conclusive part of the SR concerns the preparation, reporting and dissemination of all the results obtained, that is the documentation of the extracted data. A critical and objective analysis of the salient features of the works will be presented, comparing the results obtained and reasoning on them.

In order to disseminate the results, there are different options, which should be taken into account depending the target audience: Academic journals/conferences, practitioner-oriented magazines, posters, web pages or direct communication. The choice of the dissemination mechanism directly affects the format in which the SR will be reported. The structure and contents of this report are usually normalised in other areas [1].

The evaluation of the report will be done as an unavoidable step when it is submitted for publication to a peer-reviewed journal.

## 3. Report of the Systematic Review

This section presents the report of the systematic review on distributed estimation techniques applied to cyber-physical systems, that follows the structure presented in [1].

### 3.1. Background

**Figure 2:**
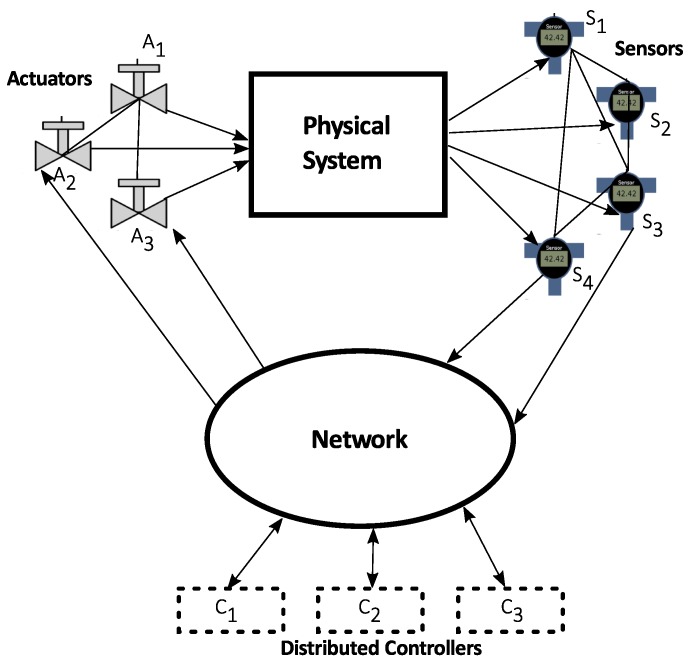
The architecture of cyber-physical systems [17].

Cyber-physical systems (CPS) integrate computing and communication capabilities with monitoring and control of entities in the physical world. These systems are usually composed by a set of networked agents including sensors, actuators, control processing units and communication devices [17], as shown in Figure 2.

One of the difficulties presented by the CPSs, which derives from their inherent complexity and heterogeneity, is the need to know or at least have a reliable estimate of the variables of the complete system, both to monitor and supervise it and to be able to control it correctly. Given that the entities that are part of a CPS have in general different sensing, computing and control capacities, as well as access to certain local information of the complete system, depending for example on their location, it is necessary to develop coordinated strategies to be able to supervise the system in a distributed way from the devices that are integrated into it. This coordination requires some sort of communication between the agents.

For the distributed estimation of the state on sensor networks there are various algorithms proposed in the literature that constitute extension of well-known classical estimators.

The first family of techniques, referred to as Kalman filters, uses a series of measurements observed over time, containing statistical noise and other uncertainties to identify the hidden state, not measurable [18]. In particular, the Kalman filter operates by propagating the mean and covariance of the state through time. In the last decades, it has been applied to distributed systems with great success, see for instance [19,20,21].

Another approach to state estimation is the Bayesian filter, which produces recursively an estimate for the targets joint probability density, given the current information [22]. It is a statistical approach to estimate, in particular for the systems that are highly nonlinear. It is a probability-based estimator [23]. Some extensions to distributed frameworks in combination with particle filters can be found in [24,25,26].

An additional noteworthy estimation technique is the Luenberger observer. It estimates the hidden internal state not measurable of a linear dynamic system from the measurements of the input and output of the system [27]. Recently, many authors have proposed different formulations of distributed Luenberger observers [28,29,30].

Further techniques exist and have found application for distributed plants. For instance, adaptive observers model the relationship between signals in real time in an iterative way, changing their coefficients according to an adaptive algorithm [31]. $H_{\infty }$ filters [23] are mainly used for multi-variable systems with couplings between the channels and with systems that have model uncertainty, see [32]. Sliding mode observers, with important measurement noise resilience, have been applied in [33]. Finally, set-membership observers are mainly used when noises and disturbances are bounded, in such a way that no statistical description is required, see [34].

### 3.2. Review Questions

The goal of this SR consists in locate and compare the different distributed estimation techniques that have been applied to cyber-physical systems. In pursuing this goal, the next research questions have been formulated:

RQ.1: What distributed estimation techniques are used in cyber-physical systems, heterogeneous systems or system of systems?

RQ.2: What are the limitations and advantages of the different techniques?

RQ.3: What are the fields of application in which these techniques are used?

RQ.3.1: In applications that include humans, animals or biological systems, which estimator obtains better results?

Since the term cyber-physical systems is kind of new, we have included the more general terms heterogeneous systems and system of systems, which sometimes are used to refer a CPS. Using the proposed PICOC criteria defined in Section 2, these research questions are described by:

Population: Cyber-physical systems, heterogeneous systems or system of systems.
Intervention: Distributed estimation.
Comparison: No additional criterion.
Outcome: Type of design (decentralised/centralised), exchanged information, communication protocol, another advantage/disadvantage.
Context: No additional criterion.

We have left the “comparison” and “context” criteria empty in order to not further restrict the search and so that more candidates’ papers are screened.

### 3.3. Review Methods

The following subsections are mainly based on the protocol presented in [35]. However, some minor modifications have been introduced since it was published. These modifications will be mentioned when appropriate.

#### 3.3.1. Data Sources and Search Strategy

We have chosen the databases presented in Table 3, namely, IEEE Xplore Digital Library, Web of Science, ScienceDirect, ACM Digital Library and Scopus. The reason for this choice is twofold. Firstly, we have excluded those databases that did not allow to make the search in the abstract, title and keywords, as detailed in Table 1. Secondly, the content of these databases covers the main publishers in automation and control, as Table 3 illustrates in a graphical way.

The time interval considered for the search is wider than the one in [35], going from 01/01/1990 to 12/09/2019.

The Boolean function for the search includes now some additional terms on top of the one in [35], since we discovered that some key primary studies were not screened. The Boolean function is, then:

(Estimator OR Estimation OR Filter OR Filtering OR Observer OR Observability OR Sensing) AND
(“Cyber Physical System” OR “Human in the loop” OR “Human Robot” OR “System of systems” OR “Heterogeneous System” OR “Human Machine” OR “Heterogeneous Multiagent System” OR “Humanoid Robot” OR “Animal Robot” “Biological System” “Physical System” “Physically-aware Engineered Systems”) AND
(Distributed OR Decentralised OR Decentralised OR “Sensor Fusion” “Multi Sensor”)

The first and third block refers to the Intervention. Note that similar terms must be included if we want to be sure that all the studies proposing distributed estimation techniques are going to be considered. The reader may find strange that the terms decentralized/decentralised or Sensor fusion appear in the search string when the proposed research questions clearly avoid them. The reason is that we have discovered that some authors use those names when they are proposing a distributed estimation technique.

In what respect to the population, the second block includes a set of key terms that are found in the literature for applications similar to those in which the SR is interested. These terms have been found by reading some survey papers in the field, with the expertise of the authors and by several post refinements after making trial searches.

#### 3.3.2. Study Selection

The guidelines presented in the protocol [35] have been followed. Two reviewers evaluate each work separately and, to be included, the primary study must be accepted by both reviewers, leaving to a third reviewer the final decision in case of disagreement. We have used the PRISMA method [9] to drive this sub-phase.

In the screening step, the inclusion and exclusion criteria are applied to the title and abstract. Then, with the preliminary candidate studies, we will perform a full-text reading to finally discard those studies that did not satisfy all the inclusion criteria.

#### 3.3.3. Study Quality Assessment

In this secluded is applied the checklist for quality assessment, show in the Table 2 to the papers selected for the SR, to highlight the quality through the different questions.

#### 3.3.4. Data Extraction and Synthesis

This SR will summarise the extracted data from the selected primary studies using a feature tables. The data that are going to be extracted from the papers and that, hence, will be included in the tables are:

- Year of publication.
- The sort of estimator used, such as a Kalman filter, a Luenberger observer, a Bayesian filter or any other.
- The application, that is, what kind of dynamical system the estimator has been applied to, such as biological systems, structural health monitoring, CPS under some kind of cyberattack and so on.
- The inclusion of simulations and/or field experiments that demonstrate the effectiveness of the used estimator.
- The estimation objective, which indicates whether the dynamics to be estimated corresponds to the whole state vector or just a partial state vector associated with a local dynamics of each subsystem.
- The design of the proposed estimator, whether it is made in a centralised or a decentralised way. Please remember that the implementation must be decentralised.
- The information that needs to be exchanged between the agents. Two aspects are mentioned here: The size of the packets to be transmitted (using state vector (n) and output or sub component of the state (r) as a reference); the frequency at which those packets must be sent, distinguishing between those estimators that require consensus steps between two consecutive sampling times and those estimators that run at the same rate as the plant.
- The communication protocol. We are not interested in the particular protocol, such as WiFi or ZigBee, but on the way each agent relates with the others. In particular, we will annotate whether the estimation algorithm requires all-to-all communication or just communication with neighboring agents. While there are other options, such as gossip or scheduled communication, no primary studies have been found to suit those categories.
- Other advantages/limitations that the authors of the primary studies mention or the reviewers discover. This feature is the only one that is subjective. However, we think that useful information that is not collected in the other features can be added at this point.

The data will be extracted by two reviewers to reduce the bias, going for a third one in case of disagreement. For the last features, both reviewers must agree on the selected advantage/limitation.

### 3.4. Included and Excluded Studies

The inclusion and exclusion criteria chosen for the selection of papers are shown in Table 4 and Table 5, respectively. We have slightly relaxed the criteria compared to the protocol in [35]. For instance, the SR now accepts papers which do not present simulations or experiments or with a length shorter than three pages.

The selected criteria ensure that the search is focused on the chosen topic. For a paper to be included, it must satisfy all the inclusion criteria and none of the exclusion criteria. As mentioned in the introduction we are interested in those heterogeneous systems that have a certain dynamic and that incorporate a physical and a cybernetic part. Furthermore, the estimators must interchange some form of information with other agents.

**Figure 3:**
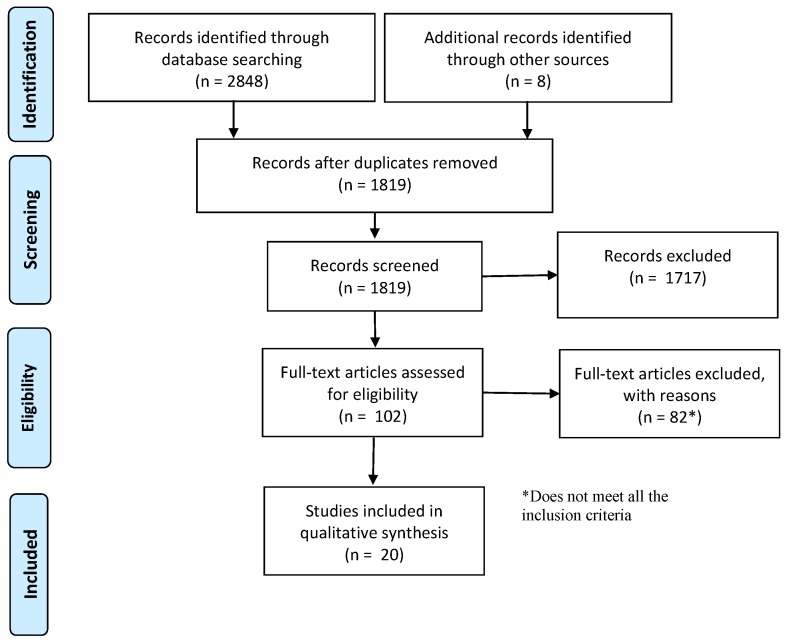
PRISMA (preferred reporting items for systematic reviews and meta-analyses) flowchart.

The number of papers obtained by launching this Boolean function in the chosen databases, in the established fields were 2848, see Table 6. With the mentioned modifications in the protocol the number of candidate studies has grown from 1788 to 2848, that is, an increment above 60%. The PRISMA complete flowchart is shown in Figure 3.

In this flowchart, in addition to the studies found in the aforementioned databases, those identified through other sources are specified, for example by contacting the authors and revising the bibliography of the selected papers. At this point the duplicates are removed, which are more than 1000 for our particular case, using an appropriate reference manager, such as Mendeley.

After the screening phase, the remaining studies are far less than the large quantity we had at the beginning: From the initial 2848 works, 102 papers remains. However, after a full-text reading, 82 papers were discarded. Finally, 20 papers have been found. In spite of the low percentage of selected papers (20 out of 2800), the analysis of these 20 papers indicates that they were an enough number of documents to identify interesting research gaps and conclusions.

### 3.5. Results

The quality of the selected papers have been assessed, obtaining the results depicted in Table 7.

The data extracted from the papers have been included in different tables, namely Table 8, Table 9, Table 10 and Table 11, each associated to a research question. In the following section, the data are analyzed and several conclusions are drawn.

### 3.6. Discussion

The systematic review is completed by carrying out the discussion. Firstly, the principal findings will be clarified and, later, an analysis on the strengths and weaknesses of the SR will be performed.

#### 3.6.1. Principal Findings

In the following, we provide a clear and detailed answer to each of the research questions that were exposed at the beginning of the review.

RQ.1: What distributed estimation techniques are used in cyber-physical systems, heterogeneous systems or system of systems?

The distributed estimation techniques used in the selected papers are varied, as Table 8 shows.

Mainly they can be divided into the following groups:

- Adaptive observer, used in [46] for multi-agent systems, in [48] for leader–follower systems and in [55] for heterogeneous multi-agent system.
- Bayesian filter, also used in many areas, such as collaborative human–robot systems [36], joint attack detection and secure state estimation [51] or 3D upper body tracking, with a combination of annealing particle filter and belief propagation inference [38].
- $H_{\infty }$ filter, which has been applied for the detection of biasing attacks on distributed estimation networks [45] and for the joint attack detection and secure state estimation [52].
- Luenberger observer, used in [40] for CPSs affected by adversarial attacks on the sensed and communicated information, in [42] for detecting and isolating multiple sensor faults, in [44] for the simultaneous estimation of the state and attack, in [53] with a secure pre-selector, in [47] for the state estimation in networks subject to adversarial attacks.
- Kalman filter, which has been used in various fields of application, such as fault detection and isolation for systems of systems [37], security of the state estimation in power systems [39], for attack detection in [54], multi-robot tracking [41], monitoring industrial CPSs [43] or estimation of the biofilm growing process in a biological system [50].

It should be mentioned the paper [49], which presents two different estimation techniques: The attack estimation is carried out by means of a $H_{\infty }$ filter, whereas the state estimation, considering the attack previously estimated, is done with a Luenberger observer.

Finally, it deserves to be remarked that some estimators have not been found to be applied to CPSs, such as sliding mode observers or set-membership observers.

RQ.2: What are the limitations and advantages of the different techniques presented in the different papers?

This question is focused on the limitations and/or advantages that have been found in the selected papers and shown in the Table 9. The answer to this question is rather complex and wide, because, when studying a paper, it is not easy to extract the limitations and/or advantages, because it is sometimes a subjective aspect. In the following, we will present some reflections and considerations extracted from the data.

Let us first pay our attention on the design of the estimators. Whereas most of the studies present design methods that can be implemented in a decentralised way, there are some papers in which the estimators need to be design in a unique centralised step. This is the case of [45,52], where a unique linear matrix inequality must be solved to find the observer gains. In [44] the authors require to solve decentralised Lyapunov matrix equations to ensure that both the state and the attack is estimated. However, those equations require global information that, in general, is not available in every location, such as the output matrices and Luenberger observer gains of every estimator.

It is worth mentioning that most authors have used $H_{\infty }$ filters for attack detection, see [45,49,52]. In order to ensure a given $H_{\infty }$ bound γ, all these papers require to solve centralised LMIs. In fact, there exists no study that has solved this problem using a pure decentralised approach. Other options available are the papers [44,51]. The former, based on a Luenberger observer, is adequate when the system is described as a set of, possibly nonlinear, subsystems. The latter uses a Bayesian filter to estimate the complete state of the plant.

Consensus algorithms have been vastly used in distributed estimation in general and in distributed estimation for CPSs in particular, see [36,37,39,40,43,45,47,49,50,51,52]. While the consensus methods are well known, it should be remarked that there are important differences in the way they influence the estimation algorithm. Mainly, we could distinguish between those consensus iterations that run at the same rate of the estimator [36,37,40,45,47,52] and those others that need to be executed many times (usually a large number of iterations, since consensus algorithms typically converge asymptotically) between two consecutive estimation steps [39,43,49,50,51]. Therefore, and despite the same word being used in the papers, enormous differences exist in what respect the information exchanged (see Table 10).

Consensus algorithms are used in those papers with several objectives. The most common application is for the agreement in the estimated state vector, see [37,39,40,45,47,52]. Another example is found in [49], where consensus is used for the residuals. The authors in [43,50] use the consensus because they need to estimate the output vector. Finally, the approaches in [36,51] incorporates a consensus algorithm to achieve an agreement in a probability density function.

Concerning the consensus gains that those algorithms use, in the vast majority of cases it consists in a scalar gain, as for example in [40,46,47,48,51,55]. In [37], a consensus matrix is proposed, but it is required to be diagonal matrix. Only the paper [45] uses a consensus matrix, but it is a unique matrix gain for every neighbor and it must be found after an LMI. Hence, it has been observed that none of the proposed studies have been able to use (and distributed design) different consensus gains for every neighbor.

To deepen the discussion concerning the required communication (see Table 10), it is noted that in those estimators based on the Luenberger observer, i.e. [40,42,44,47,53], the information exchanges between agents, take place at the same rate as the estimation algorithm. Moreover, the agents exchange the estimated state vector or a sub component of it. On the contrary, those approaches based on Kalman filters usually require a lot of information, as in [43], or consensus iterations between estimation steps, as in [39,50], with the consequent communication effort. A similar drawback appears in [38], where a lot of information must be sent between the particles of the filter between two consecutive sampling instants.

Distributed estimation techniques have been also applied to fault detection and isolation, see [37,42]. Both studies present distributed fault detection algorithms, for LTI systems [37] and for Lipschitz nonlinear systems [42]. It should be noted that, whereas in [42] a fusion center is required for fault isolation, the algorithm presented in [37] is able to provide distributed decisions.

Finally, it is worth mentioning the research developed in [36,41] for heterogeneous multi-agent systems. These studies consider a group of robots that are endowed, among others, with an estimation unit. These units have the objective of estimating the state (position, velocity orientation) of the associated robot and some target (a ball position, as in [41] and other robots’ states, as in [36]). The presented estimation algorithms can be implemented in moving agents.

RQ.3: What are the fields of application in which these techniques are used?

Most of the applications found in the selected papers lie within the following four main categories (see Table 11):

- Heterogeneous multi-agent system: Different cases are considered in these studies in which there are different types of systems. In [36] a scalable collaborative human–robot system for information gathering applications, through a decentralized Bayesian fusion algorithm, is presented. The results of a collaborative multi-target search experiment conducted with a team of four autonomous mobile sensor platforms and five humans carrying small portable computers with wireless communication are presented to demonstrate the efficiency of the approach. In the paper [41], a multi-object, multi-sensor and cooperative tracking method using a Kalman filter is proposed for the Robocup Standard Platform League, where two teams of humanoid robots play soccer against each other.

It is worth mentioning that the documents [46,48,55] deal with the same application, that is, the synchronization of heterogeneous systems. All those papers propose the use of an adaptive observer, with different modifications, as will be mentioned next.

The exogenous signal representing the reference input to be tracked is assumed to be generated by a so-called exosystem as follows:

(1) $${\dot{x}}_{0}\left(t\right)=S_{0}x_{0}\left(t\right),$$

with $S_{0}$ being a known constant matrix.

The agents are modelled as linear time-invariant systems described by:

(2) $$\begin{array}{c} \begin{array}{cc} {\dot{x}}_{i}\left(t\right) & =A_{i}x_{i}\left(t\right)+B_{i}u_{i}\left(t\right)+E_{xi}x_{0}\left(t\right)+E_{wi}w_{i}\left(t\right),\\ y_{mi}\left(t\right) & =C_{mi}x_{i}\left(t\right)+D_{mi}u_{i}\left(t\right)+F_{m_{xi}}x_{0}\left(t\right)+F_{m_{wi}}w_{i}\left(t\right), \end{array} \end{array}$$

where for i=1,⋯,N, $x_{i}$, $y_{mi}$, $u_{i}$ are the state, measurement output and input of the ith subsystem. External disturbances $w_{i}$ are assumed to be generated by an independent linear system, that is, $\dot{w_{i}}\left(t\right)=Q_{i}w_{i}\left(t\right)$.

The authors in [46] propose a self-tuning observer to estimate the state of the leader from each agent. Then, using this information, they compute appropriate control inputs to achieve the synchronization between the states of the leader and followers.

The main novelty of [48] is that both the leader’s and the follower’s dynamics are assumed to be unknown. On the other hand, the synchronization problem is posed in [55] as a distributed optimal tracking problem, deriving inhomogeneous algebraic Riccati equations to solve it.

- Attack detection and secure estimation: Intense research has been done in these categories, with some papers tackling both challenges at the same time. They represent most of the applications encountered, even if in some papers it is only the attack detection, like in [45,49,54] and in others only the secure estimation, like in [39,40,47]. Only in [44,51,52,53] are both considered. Secure estimation is certainly among the most addressed topics in the reviewed articles. Perhaps the main difference between those papers is the typology of the attacks/attackers and the way the secure estimation is achieved.

**False data injection attack:** This is when a malicious adversary launches false data injection attacks at the physical system layer to intentionally modify the system’s state and/or the measured output. From a mathematical point of view, false data injection attacks are usually modeled as additive disturbance or additive noise:

(3) $$x^{+}=\left\{\begin{array}{cc} f_{1}\left(x,u,w\right), & \mathrm{Under}\mathrm{no}\mathrm{attack}\\ f_{2}\left(x,u,w,a\right), & \mathrm{Under}\mathrm{attack} \end{array}\right.,\;y=\left\{\begin{array}{cc} h_{1}\left(x,v\right), & \mathrm{Under}\mathrm{no}\mathrm{attack}\\ h_{2}\left(x,v,b\right), & \mathrm{Under}\mathrm{attack} \end{array}\right.,$$

with x,u,w,v being the state, input, external disturbance and measurement noise, respectively, and a,b the false data injection in the system dynamics or the measured output.

This is the case in [44], in which the attacks affects both the dynamics of a nonlinear descriptor system and the measurement output. This paper proposes a distributed robust approach using a Luenberger observer. Similarly, in [51], the attack detection-state estimation problem is formulated in the context of random set theory by representing the joint information on the attack presence/absence, on the system state and on the signal attack, in terms of a hybrid Bernoulli random set density, using a recursive Bayesian filter.

Several authors consider a simplification of the previous problem by assuming a linear time-invariant system, such as:

(4) $$\begin{array}{c} \begin{array}{cc} x(k+1) & =Ax\left(k\right)+Bu\left(k\right)+B_{w}w\left(k\right)+Ea\left(k\right),\\ y(k) & =Cx(k)+v(k)+b(k), \end{array} \end{array}$$

where x(k),u(k),y(k),w(k),v(k) are, respectively, the state, input, the measurement output, disturbances and the process noise at the k-th time step. The attack signals are a(k),b(k).

In this line, [52] studies the situation in which the attacks only affects the system dynamics (i.e., b(k)≡0). They propose an distributed $H_{\infty }$ filter for attack detection and secure estimation. The attacks on the outputs are analyzed in [53,54] with a distributed Kalman filter in the former and a Luenberger observer in the latter. Finally, [49] considers a descriptor linear time-invariant system whose outputs can be compromised by false data injection attacks. The authors combine a Luenberger observer and an $H_{\infty }$ filter to detect the attack and be resilient to its effect.

It is worth mentioning the work [45], which studies a different situation in which the attacks directly affect the estimator dynamics:

(5) $${\dot{\hat{x}}}_{i}\left(t\right)=Ax_{i}\left(t\right)+L_{i}\left(y_{i}\left(t\right)-C_{i}{\hat{x}}_{i}\left(t\right)\right)+K_{i}\sum_{j}\left({\hat{x}}_{j}\left(t\right)-{\hat{x}}_{i}\left(t\right)\right)+f_{i}\left(t\right),$$

with $f_{i}\left(t\right)$ the signal attack to be detected using, in this case, a distributed $H_{\infty }$ filter.

**Jamming attack:** This sort of attack pursues to block the wireless transmission channels between sensors and remote estimators, incurring in a possible packet loss or a partial degradation of the information. Therefore, it is considered a situation in which the sensors are not physically located near the agent, unlike the previous cases.

To model jamming attacks, the authors in [52] use the following formulation:

(6) $${\hat{y}}_{i}\left(k\right)=y_{i}^{attk}\left(k\right)+y_{i}^{comp}\left(k\right)+D_{i}v_{i}\left(k\right),\;\forall i,$$

where the corrupted measurement ${\hat{y}}_{i}\left(k\right)$ that the remote estimator receives consists of two terms plus a noise:

(7) $$\begin{array}{c} \begin{array}{cc} y_{i}^{attk}\left(k\right) & =\theta_{i}\left(k\right)y_{i}\left(k\right),\\ y_{i}^{comp}\left(k\right) & =\left(1-\theta_{i}\left(k\right)\right){\hat{y}}_{i}\left(k-1\right), \end{array} \end{array}$$

and specifically:

- $y_{i}^{attk}\left(k\right)$ stands for the attacked and manipulated measurement term. Signal $y_{i}\left(k\right)$ is the actual output measured by the sensor.
- $y_{i}^{comp}\left(k\right)$ represents the compensated measurement term corresponding to the lossy measurement $y_{i}^{attk}\left(k\right)$ caused by the attacker.
- $v_{i}\left(k\right)$ denotes the measurement noise experienced through the wireless channel.

The stochastic variable $\theta_{i}\left(k\right)$ takes values of 1 and 0, with $\theta_{i}\left(k\right)=0$ representing the case of a jamming attack.

With the assumption of $\theta_{i}\left(k\right)$ being a Bernoulli distributed white sequence, with known expected value $E\left\{\theta_{i}\left(k\right)\right\}=\beta_{i}$, a measurement compensation unit is proposed in [52] for this kind of attack. It is based on a buffer that stores past data that are used in case of attacks.

To tackle the random jamming attacks, a refined measurement output model based on compensated measurements has been proposed and resilient estimators have been constructed.

**Fake communications:** with this name, we refer the situation in which the attacker is able to gain control of a communication link to substitute real packets or to inject extra packets when two agents or estimators interchange some sort of information. It is, therefore, an attack that takes place in the cyber layer. Secure estimators for this sort of attacks is presented in [49,51,54].

Denoting by $Z_{i,j}\left(k\right)$ the information that agent *i* receives from agent *j*, fake communication are modeled in [51] as:

(8) $$Z_{i,j}\left(k\right)=Y_{i,j}\left(k\right)\cup F_{i,j}\left(k\right),$$

where

$$Y_{i,j}\left(k\right)=\left\{\begin{array}{cc} y_{i,j}\left(k\right) & \mathrm{no}\mathrm{attack}\\ 0 & \mathrm{complete}\mathrm{packet}\mathrm{substitution}\\ {\tilde{y}}_{i,j}\left(k\right) & \mathrm{packet}\mathrm{modification} \end{array}\right.,$$

and $F_{i,j}\left(k\right)$ is the set of any fake packet originated by the attacker.

In particular, the paper [51] analyses a cluster-based network, wherein multiple cluster-heads receive data from remote sensors via non-secure links and exchange processed information neighborwise via secure links.

Fake communications are also considered in [49], in which the authors introduce a specific cyber-attack on the communication links between monitoring centres, in addition to false data injection sensor attacks. The novel Kullback-Liebler divergence based detector is used in [54] to capture the fake communications.

**Fake agent:** This is the case where either the attacker has gained control of an agent or the agent itself is the attacker. In both cases, the information that this agent sends to the rest of estimators can be compromised.

A trust-based mechanism is proposed in [39,40] to cooperatively detect the fake agent and reduce the impact of the information received from it, in the first case through a Kalman filter, while in the second with a Luenberger observer. A similar version of the fake agent can be found in [47] under the name of Byzantine adversaries. In this case, the problem is analyzed using a distributed Luenberger observer based on subspace decomposition. This kind of Byzantine agent is given complete knowledge of the network and system dynamics and is allowed to deviate from the rules of any prescribed algorithm. Sufficient conditions for state estimation are provided in [47], relying on the construction of a directed acyclic graph. In this paper, the authors allow for the possibility that certain nodes in the network are compromised by an adversary and do not follow their prescribed state estimate update rule.

Two subsets of V (set of nodes) are created: R comprising of regular nodes and A=V\R comprising of adversarial nodes. They consider the Byzantine fault model where an adversarial node can arbitrarily deviate from the rules of any prescribed algorithm and can transmit different state estimates to different neighbors at the same time step. In addition, the adversarial nodes possess complete knowledge about the graph topology and the plant dynamics, i.e., an adversarial node knows the measurements of the normal nodes at every time step. They endow such privileges to the adversaries with the aim of providing resilience to worst-case behavior. This is known in the literature, as the f-total adversarial model.

**Definition** **1.** *(Omniscience) A distributed observer achieves omniscience if $\lim_{k\to \infty }∥{\hat{x}}_{i}\left(k\right)-x\left(k\right)∥=0,\;\forall i\in \left\{1,\cdots ,N\right\}$, i.e., the state estimate maintained by each node asymptotically converges to the true state of the plant.*

**Definition** **2.** *(f-local set) A set C ⊂ V is f-local if it contains at most f nodes in the neighborhood of the other nodes, i.e., |N∩C|≤f,∀i∈V\C.*

**Definition** **3.** *(f-local adversarial model) A set A of adversarial nodes is f-locally bounded if A is an f-local set.*

Considering the following system (Equation (9)):

(9) $$\begin{array}{c} \begin{array}{cc} x(k+1) & =Ax(k),\\ y_{i}\left(k\right) & =C_{i}x\left(k\right), \end{array} \end{array}$$

the problem in [47], is to formulate a state estimation scheme so that

(10) $$\lim_{k\to \infty }∥{\hat{x}}_{i}\left(k\right)-x\left(k\right)∥=0\;\forall i$$

regardless of the actions of any f-locally bounded set of Byzantine adversaries.

- Fault detection and isolation (FDI), are found in [37,42]. In [37] a new algorithm is proposed for distributed fault detection and isolation, applicable to systems of systems based on Kalman filter. The mathematical formulation for a fault detection and isolation system with *q* faults presented in [37] is:
  (11) $$\begin{array}{c} \begin{array}{cc} \dot{x}\left(t\right) & =Ax\left(t\right)+\Gamma w\left(t\right)+\sum_{k=1}^{q}{\bar{F}}_{k}{\bar{\mu }}_{k}\left(t\right),\\ y(t) & =Cx(t)+v(t), \end{array} \end{array}$$
  where *x*, *y*, *w*, *v* are the state, output, input and measurement noise vectors, respectively, of the system.

The authors assume that there is one target fault to be detected by every agent i∈1,⋯,q, denoted $\mu_{1}\left(t\right)={\bar{\mu }}_{i}\left(t\right)$; the rest of the faults represent the nuisance fault $\mu_{2}$, i.e., $\sum_{k=1}^{q}{\bar{F}}_{k}{\bar{\mu }}_{k}\left(t\right)=F_{1}\mu_{1}\left(t\right)+F_{2}\mu_{2}\left(t\right)$.

The main contribution of [42] is the design and analysis of a fault diagnosis methodology, with emphasis on the distributed isolation of multiple sensor faults that may affect the physical part of multiple interconnected cyber-physical systems, which may exchange sensor information related to the physical interconnections. This methodology builds upon a distributed Luenberger observer.

- Other applications: In this group, three particular studies not related to the previous groups were included. In [38], the authors propose a new approach for 3D upper body pose estimation, using a combination of an annealing particle filter and belief propagation inference. The work [43] is concerned with the distributed estimation problem, adopting a Kalman-like filter, for industrial automation over relay assisted wireless sensor networks. Finally, in [50] a biological system is considered. In this work, the authors present a framework for the use of a wireless sensor network as an estimator of the biofilm evolution in a reverse osmosis membrane so that effective solutions can be applied before the irreversible phase is attained.

RQ.3.1: In applications that include humans, animals or biological systems, which estimator obtains better results?

While the review has been targeted at this kind of application, not many items have been found that include humans, animals or biological systems. Consequently, it is not possible to make real comparisons or to extract the best conclusions. For instance, no paper was found that includes animals in the application.

We must firstly mention [50], which uses a distributed Kalman filter for a biological system. In particular, the authors present a deployment of a wireless sensor network to estimate the biofilm evolution in a reverse osmosis membrane. They obtain nice results in simulation. However, in order to extract valuable conclusions, experimental validation is required.

Another paper that satisfies this requirement is [36]. It presents a coordinated network of humans and robots for state estimation using a Bayesian filter. Peer-to-peer collaboration between human–computer augmented nodes and autonomous mobile sensor platforms happens by sharing information via wireless communication network. The proposed method is tested with experiments, which show that improved results are obtained due to the human–robot collaboration.

#### 3.6.2. Strengths and Weaknesses

This study presents several weaknesses, some that are typically common to all SRs and others that appear due to the inexistence of this kind of review applied to our field:

- With the aforementioned criteria, five digital databases have been chosen to include as many relevant papers as possible. However, all published papers on the topic cannot be analyzed, limiting the review conducted.
- Another possible weakness might be the inclusion and exclusion criteria adopted for selecting papers. For example, we have focused on papers published in English, but there might be relevant studies written in other languages.
- Many databases are not prepared for such an accurate research as the one described in Section 3.3.
- There exists no normalisation for the contents of abstract and title in control and automation journals and conferences, a problem shared with other fields, such as computer science. Moreover, although the keywords are sometimes normalised, they are not usually peer-reviewed. This makes it difficult to make a correct screened reading just title and abstract. Then, full text must be revised, so the time devoted in the very first phases of the review is really large. In contrast, the abstract of many social science papers must include, for instance, explicit reference to objectives, methods, results and conclusions; this eases the screening process.
- When extracting the data, it is often harder to compare two techniques or two aspects treated differently to in other sectors, such as medicine or psychology, where mainly studies and/or clinical data are compared. Then, we are forced to make a qualitative rather than a quantitative comparison (for instance, a meta-analysis). A possible solution to this weakness would be the formulation of benchmark problems, in which the same problem would be faced with different approaches, so as to be able to extract adequate quantitative conclusions.

On the other hand, this systematic review presents some strengths that must be pointed out:

- From the authors’ knowledge, this is the first systematic review in automation and control, inheriting the good practices in areas in which these reviews are a common practice.
- The study follows the PRISMA guidelines for reporting systematic reviews to meet the highest quality. As a consequence, the SR identifies all the relevant works produced on the topic following an explicit and reproducible research methodology.
- Some recommendations for the control community have been proposed: Normalisation in the information included in the title, abstract and keywords; definition of benchmarks problems to make qualitative comparisons of different estimation techniques; or development of better search engines in the databases, just to mention some of them.

#### 3.6.3. Detected Gaps and Future Research

The SR has discovered important gaps that should be filled in the following years:

- Pure distributed $H_{\infty }$-based attack detection mechanism: Both papers that incorporate an $H_{\infty }$-filter for attack detection require a centralized design based on LMIs [45,52]. Providing a decentralized mechanism for the observer synthesis seems to be compulsory, specially in these frameworks in which a cyber-attack is able to disable an agent or its communications.
- Inclusion of matrix gains for consensus-based estimators: Scalar gains [40,46,47,48,51,55] and diagonal matrices [37] have been proposed in the literature to weight the consensus agreement. Using a complete matrix would be useful to differentiate the weights of the different components in the consensus. Decentralized design of these matrices must also be pursued. In fact, in [45] the authors propose the use of a consensus matrix, but it is common for all the agents and must be found by solving a centralized LMI.
- Modifications of the distributed Kalman filter with reduced communication effort: Either because of the consensus iterations or the need of additional matrices (covariance or information matrices), the proposed DKF are the ones that rely on heavier communication effort to fulfill their objectives. Moving to DKF formulations in which just a subset of the complete state vector is sent should be an interesting goal for the next years.
- Privacy and security in CPS: Some of the primary studies deal with the problem of secure estimation in the presence of attacks (see Table 11). However, nothing has been said about the privacy of the information exchanged between agents. Coping with these issues in an open environment, in which the wireless communications can suffer from any malicious action, must also be analyzed in depth in the future.
- Lack of sliding mode observers and guaranteed estimators for CPS: None of the studies have used this kind of observer structure for the estimation of CPS. While they are very common in the literature, the SR concludes that more effort is necessary in the adaptation of those methodologies that have shown great success in the estimation of nonlinear models.
- Practical absence of estimators for biological, human and animal environments: As examined through the research question 3.1 only two studies have been found with this kind of application. This reveals, in the authors’ opinion, that the background of the researchers in estimation of CPS is greater in the cybernetic layer than in the physical one. Increased multi-disciplinary research must be done to fill this gap.
- Almost inexistence of complex models, such as hybrid systems, to describe the physical part of the CPS: While the modelling of complex systems has been the subject of intense research, this SR discloses that those formulations have not been used when the problem of distributed estimation of CPS is to be solved. In fact, most of the studies use linear models [37,40,43,45,46,47,48,49]. Unfortunately, the inclusion of external human commands or the occurence of a discrete event, are better captured with other structures.

In addition, the SR has presented some recommendations to the community, including editors or databases, that were mentioned before.

## 4. Conclusions

This paper presents a systematic review of the distributed estimation techniques applied to CPS, system of systems and heterogeneous systems that have been published in the period of time from the early 1990s to September 2019. Prior to the report of the SR, a brief presentation of the whole procedure of an SR adapted to our field has been presented.

The studies that have been included have been analyzed to respond to the research questions posed, that is, what are the techniques applied, what are their advantages and limitations and in which field have they been applied. These results have been included in several tables to illustrate the findings. Moreover, the SR has detected existing gaps in the literature and proposed future research lines to the community.

## Figures and Tables

**Table 1:** Some of the most important databases in automation and control: A = abstract, T = article title, K = keywords, F = full text.

Databases	Search Fields	Manual or Automatic Search	Supported Operators	$N^{o}$ Terms Supported	$N^{o}$ Maximum Download	Citations Format	Download with “abstract"
Web of Science	A+T+K, T, F	Both	AND, OR, NOT, NEAR, (), *, “"	Not specified	50 citations	bib, RIS, CSV	YES
IEEE Xplore	A+T+K, A, T, K, F	Both	AND, OR, NOT, NEAR, (), *, “"	Only 40	2000 citations	bib, RIS, CSV	YES
ScienceDirect	A+T+K, A, T, K, F	Both	AND, OR, AND NOT, (), *, ?, “", {}	Not specified	200 citations	bib, RIS, Text	YES
ACM Digital Library	A+T+K, A, T, K, F	Both	AND, OR, NOT, (), “”	Not specified	2000 citations	bib, RIS, CSV	NO
Scopus	A+T+K, A, T, K, F	Both	AND, OR, AND NOT, *, ?, “”, ()	Not specified	2000 citations	bib, RIS, CSV, Text	YES
SpringerLink	T, F	Automatic	AND, OR, NOT, “”, ()	Not specified	2000 citations	CSV	NO
Wiley Online Library	A, T, K, F	Automatic	AND, OR, NOT, “”, *, ()	Not specified	20 citations	bib, RIS, Text	YES
Google Scholar	T, F	Automatic	AND, OR, NOT, “”, ()	Not specified	1 citation	bib, RIS	NO

**Table 2:** Checklist for quality assessment.

	Question	Score
Q1	Is the problem presented clearly?	Yes/Partially/No
Q2	Is the methodology used presented clearly?	Yes/Partially/No
Q3	Are there any limitations and/or restrictions?	Hard/Soft/No
Q4	Is there a discussion of the results?	Yes/Partially/No
Q5	Does it answer all the questions originally formulated by the SR?	Yes/Partially/No
Q6	Has it been cited by many authors?	Cites/Year
Q7	Has it been published in a journal or conference proceeding?	Journal/Conference

**Table 3:** Databases coverage with respect to the content of the publishers [35].

	IEEE Xplore	ACM Digital Library	Scopus	Web of Science	Science Direct
IEEE					
IET					
Pegamon Elsevier					
Elsevier Science					
Wiley Blackwell					
Taylor and Francis					
Springer					
SIAM Publications					
Oxford University Press					
Korean Inst. Electrical Eng.					
Sage Publications					
ASME					
Microtome Publications					

**Table 4:** Inclusion criteria.

Full paper available online (through search engines or by contacting the authors) Use or propose a distributed estimationtechnique on cyber-physical systems, heterogeneous systems or system of systems or make specific reference to humans, animals or biological systems Use a distributed estimator with some sort of communication between local estimators The system to be estimated must have dynamics

**Table 5:** Exclusion criteria.

Secondary studies and gray literature Non-English written papers Duplicated studies Studies clearly irrelevant to the research Focused only on control

**Table 6:** Studies obtained by each database.

Database	Studies
Web of Science (WoS)	571
IEEE Xplore (IEEEX)	919
ScienceDirect (SD)	51
ACM Digital Library (ACM)	525
Scopus	782
	2848

**Table 7:** Checklist for quality assessment.

	Q1	Q2	Q3	Q4	Q5	Q6	Q7
[36]	Yes	Yes	No	Yes	Partially	27/2008	Conference
[37]	Yes	Partially	Soft	No	Yes	2/2010	Conference
[38]	Yes	Yes	Soft	Yes	Yes	1/2012	Journal
[39]	Yes	Partially	Soft	Partially	Yes	24/2012	Conference
[40]	Yes	Partially	Soft	Yes	Yes	17/2013	Conference
[41]	Yes	No	No	Yes	Yes	5/2013	Conference
[42]	Yes	Yes	No	Yes	Yes	46/2014	Journal
[43]	Yes	Yes	Soft	Yes	Yes	81/2015	Journal
[44]	Yes	Yes	Hard	Partially	Yes	3/2016	Conference
[45]	Yes	Yes	Hard	No	Yes	9/2016	Conference
[46]	Yes	Yes	Soft	Partially	Yes	5/2016	Conference
[47]	Yes	Yes	Soft	Partially	Yes	25/2016	Conference
[48]	Yes	Yes	Soft	Yes	Yes	47/2016	Journal
[49]	Yes	Partially	Soft	Partially	Yes	2/2017	Conference
[50]	Yes	Yes	Soft	Partially	Yes	01/2017	Conference
[51]	Yes	Yes	No	Yes	Yes	14/2018	Journal
[52]	Yes	Yes	Hard	Yes	Yes	38/2018	Journal
[53]	Yes	Yes	Soft	Yes	Yes	1/2018	Journal
[54]	Yes	Yes	No	Yes	Yes	1/2018	Conference
[55]	Yes	Yes	Soft	Yes	Yes	13/2018	Journal

**Table 8:** Feature table. Estimation technique and application.

Cite	Year	Estimator Used	Application
[46]	2016	Adaptive observer	Heterogeneous multi-agent system
[48]	2016	Adaptive observer	Heterogeneous multi-agent system
[55]	2018	Adaptive observer	Heterogeneous multi-agent system
[36]	2008	Bayesian filter	Heterogeneous multi-agent system
[51]	2017	Bayesian filter	Attack detection and secure estimation
[38]	2012	Particle filter + Belief Propagation inference	3D Upper body pose estimation
[52]	2017	$H_{\infty }$ filter	Attack detection and secure estimation
[45]	2016	$H_{\infty }$ filter	Attack detection
[49]	2017	Luenberger observer and $H_{\infty }$ filter	Attack detection
[40]	2013	Luenberger observer	Secure estimation
[47]	2016	Luenberger observer	Secure estimation
[42]	2014	Luenberger observer	Fault detection and isolation
[44]	2016	Luenberger observer	Attack detection and secure estimation
[53]	2018	Luenberger observer with secure pre-selector	Attack detection and secure estimation
[37]	2010	Kalman filter	Fault detection and isolation
[39]	2012	Kalman filter	Secure estimation
[41]	2013	Kalman filter	Heterogeneous multi-agent system
[43]	2015	Kalman filter	Monitoring industrial CPSs
[50]	2017	Kalman filter	Biological system
[54]	2018	Kalman filter	Attack detection

**Table 9:** Features table. Limitations and advantages according to a given criteria. Color code: Green = desired, yellow = intermediate, red = undesired.

Cite	Estimator Used	Experiment or Simulation	Estimation Objective	Design	Exchanged Information	Communication Protocol
[46]	Adaptive observer	Simulation	Local state	Decentralised	Estimated state vector (n) + adaptive system matrix (n*n), same rate as the system	Neighbourhood
[48]	Adaptive observer	Simulation	Local state	Decentralised	Estimated state vector (n) + adaptive system matrix (n*n), same rate as the system	Neighbourhood
[55]	Adaptive observer	Simulation	Local state	Decentralised	Estimated state vector(n) + adaptive system matrix (n*n), same rate as the system	Neighbourhood
[36]	Bayesian filtering	Experiment	Complete state	Decentralised	Estimated state vector (n) and output (r<n), same rate as the system	Neighbourhood
[51]	Bayesian filter	Simulation	Complete state	Decentralised	Estimated state vector (n) consensus between sampling instants	Neighbourhood
[38]	Particle filter + Belief Propaga-tion inference	Experiment	Local state	Decentralised	Estimated state vector (n) * Number of particles (N), at a rate $N_{BP}$ higher than the rate of the system	Neighbourhood
[52]	$H_{\infty }$ filter	Simulation	Complete state	Centralised	Estimated state vector (n), same rate as the system	Neighbourhood
[45]	$H_{\infty }$ filter	None	Complete state	Centralised	Estimated state vector (n), same rate as the system	Neighbourhood
[49]	Luenberger observer and $H_{\infty }$ filter	Simulation	Local state	Decentralised	Estimated state vector (n), consensus between sampling instants	Neighbourhood
[40]	Luenberger observer	Simulation	Complete state	Decentralised	Estimated state vector (n) and output (r<n), same rate as the system	Neighbourhood
[47]	Luenberger observer	None	Complete state	Decentralised	Estimated state vector (n), same rate as the system	Neighbourhood
[42]	Luenberger observer	Simulation	Local state	Decentralised	Subset of the estimated state vector (r<n), same rate as the system	Neighbourhood
[44]	Luenberger observer	Simulation	Local state	Centralised	Estimated state vector (n), same rate as the system	All-to-all
[53]	Luenberger observer with a secure pre-selector	Simulation	Local state	Decentralised	Estimated state vector (n), same rate as the system	All-to-all
[37]	Kalman filter	Simulation	Complete state	Decentralised	Estimated state vector (n) and residuals (n), same rate as the system	All-to-all
[39]	Kalman filter	Simulation	Complete state	Decentralised	Estimated state vector (n), consensus between sampling instants	Neighbourhood
[41]	Kalman filter	Both	Local state	Decentralised	Estimated state vector (n), same rate as the system	All-to-all
[43]	Kalman filter	Simulation	Complete state	Decentralised	Estimated state vector (n), same rate as the system + augmented output vector (n) and augmented noise matrix (n*n), tree-based broadcasting + augmented output vector	Neighbourhood
[50]	Kalman filter	Simulation	Complete state	Decentralised	Estimated state vector (n), consensus between sampling instants	Neighbourhood
[54]	Kalman filter	Simulation	Local state	Decentralised	Estimated state vector (n) same rate as the system	Neighbourhood

**Table 10:** Features table. Other limitations and advantages.

Cite	Estimator Used	Limitations	Advantages
[46]	Adaptive observer	Scalar gains. Measure the whole state	Do not require to know the system matrix. The consensus gains are dynamically chosen
[48]	Adaptive observer	Scalar gains.	Do not require to know the system matrix
[55]	Adaptive observer	Scalar gains. Leader’s dynamics is required	Do not require to know the system matrix
[36]	Bayesian filter	Acyclic communication graphs	Moving sensors and targets. Collaboration between human and robots. Consider packet dropouts
[51]	Bayesian filter	Secure communication between fusion nodes. Scalar consensus gains	Nonlinear systems. Different kinds of attacks
[38]	Particle filter + Belief Propagation inference	Communication effort	Linear complexity according to the number of body parts
[52]	$H_{\infty }$ filter	LMI centralised design. Require statistical information	Different kinds of attacks.
[45]	$H_{\infty }$ filter	LMI centralised design	Local and consensus matrix gains Attacks on the estimator dynamics
[49]	Luenberger observer and $H_{\infty }$ filter	No method for design the observer gains. Centralised detection based on $H_{\infty }$ filter. Communication effort	Descriptor system. Attack policy on sensor signals
[40]	Luenberger observer	Direct state observations. Full rank output matrix. Scalar consensus gains	Robust against compromised communication
[47]	Luenberger observer	Constraints in the system matrix. Scalar consensus gains	Decoupling of observable and unobserval dynamics. Byzantine adversaries
[42]	Luenberger observer	Fusion center for fault isolation	Observer for Lipschitz nonlinear systems. Multiple fault detection and isolation. Structured fault sensitivity
[44]	Luenberger observer	Global information for design	Nonlinear descriptor systems. Neural network for uncertainty approximation
[53]	Luenberger observer with a secure pre-selector	Only out of sensors are manipulated arbitrarily by attackers	the exact secure state estimation is achieved in a pregiven finite time
[37]	Kalman filter	The consensus matrix gains are diagonal. There is no algorithm to design these gains	Distributed decision make without fusion center.
[39]	Kalman filter	Communication effort. Consensus constraints as in Olfati Saber [19]	Robust against false data injection
[41]	Kalman filter	The estimator is not presented formally	Moving sensors. Low computational requirements
[43]	Kalman filter	Communication effort	Two kinds of nodes: sensor and relay nodes
[50]	Kalman filter	Mono-variable system. Requires statistical information of the graph. Communication effort	Consensus under interferences, packet losses and different topologies
[54]	Kalman filter	Communication effort	There are no restrictions on the types of attacks

**Table 11:** Features table. Fields of application of the surveyed studies.

Application	Studies
Heterogeneous multi-agent system	[36,41,46,48,55]
Fault detection and isolation	[37,42]
Attack detection	[44,45,49,51,52,53,54]
Secure estimation	[39,40,44,47,51,52,53]
Others	[38] 3D Upper body pose estimation
	[43] Monitoring industrial CPSs
	[50] Biological system
